# Association between periodontal disease and non-communicable diseases: A 12-year longitudinal health-examinee cohort study in South Korea

**DOI:** 10.1097/MD.0000000000007398

**Published:** 2017-06-30

**Authors:** Jae-Hong Lee, Jin-Young Oh, Tae-Mi Youk, Seong-Nyum Jeong, Young-Taek Kim, Seong-Ho Choi

**Affiliations:** a Department of Periodontology, Daejeon Dental Hospital, Wonkwang University College of Dentistry, Daejeon; b Department of Periodontology, Research Institute for Periodontal Regeneration, Yonsei University College of Dentistry, Seoul; c Department of Health Insurance Research; d Department of Periodontology, Ilsan Hospital, National Health Insurance Service, Goyang, Korea.

**Keywords:** cohort analysis, non-communicable diseases, periodontal disease, retrospective study

## Abstract

The National Health Insurance Service–Health Examinee Cohort during 2002 to 2013 was used to investigate the associations between periodontal disease (PD) and the following non-communicable diseases (NCDs): hypertension, diabetes mellitus, osteoporosis, cerebral infarction, angina pectoris, myocardial infarction, and obesity.

Univariate and multivariate logistic regression analyses adjusting for potential confounders during the follow-up period—including age, sex, household income, insurance status, residence area, health status, and comorbidities—were used to estimated odds ratios (ORs) with 95% confidence intervals (CIs) in order to assess the associations between PD and NCDs.

We enrolled 200,026 patients with PD and 154,824 subjects with a healthy oral status. Statistically, significant associations were found between PD and the investigated NCDs except for cerebral and myocardial infarction after adjusting for sociodemographic and comorbidity factors (*P* < .05). In particular, obesity (OR = 1.30, 95% CI = 1.04–1.63, *P* = .022), osteoporosis (OR = 1.22, 95% CI = 1.18–1.27, *P* < .001), and angina pectoris (OR = 1.22, 95% CI = 1.17–1.27, *P* < .001) were significantly and positively associated with PD.

This longitudinal cohort study has provided evidence that patients with PD are at increased risk of NCDs. Further studies are required to confirm the reliability of this association and elucidate the role of the inflammatory pathway in periodontitis pathogenesis as a triggering and mediating mechanism.

## 1 Introduction

Acute and chronic periodontal diseases (PDs) are considered the most common cause of loss of teeth and periodontal tissues such as the cementum, periodontal ligament, and supporting alveolar bone in adults.^[1]^ In 2015, the Health Insurance Review and Assessment Service (HIRA) reported that PD is the most costly health condition (about US$ 900 million) and second-most-frequent disease (about 26.3% [13 million] of the Korean population) among outpatients in South Korea.^[2]^ Many recent studies have demonstrated significant positive correlations between PD and various systemic disease processes.^[3,4]^ Preventing and treating PD, therefore, influences the quality of life of many people.^[5]^

Interest in non-communicable diseases (NCDs) is increasing, and the WHO reported that NCDs cause over 38 million deaths worldwide in 2012.^[6]^ NCDs including cardiovascular disease, cancer, chronic respiratory disease, diabetes, Alzheimer's disease, and osteoporosis are chronic and noninfectious disorders that are likely to progress slowly over many years.^[7]^ The National Statistical Office reported that the mean life expectancy of the Korean population in 2014 was 82.4 years. This is higher than the mean in most other countries of the Organization for Economic Cooperation and Development, and is increasing continuously. In contrast, the health life expectancy decreased to below 66 years (65.4 years) in 2012. The gap of 17 years between the mean life expectancy and healthy life expectancy may be at least partially attributable to physical damage, but NCDs have also been reported to be major causes of this discrepancy. The prevalence of NCDs has increased steadily due to increases in smoking and alcohol consumption, lack of physical activity and exercise, and poor dietary habits, and NCDs accounted for 77.4% of the direct causes of deaths among the Korean population in 2012.^[8]^

PD and NCDs are worldwide representative chronic disorders that share many risk factors such as older age, active smoking, stress, and uncontrolled blood pressure and glucose level.^[9]^ The mechanisms underlying diverse systemic diseases including PD and NCDs have been identified and reported through systematic reviews of the experimental and epidemiological evidence.^[10,11]^ Despite PD being a chronic bacterial infectious disease, a new strategy aimed at preventing and treating PD is associated with a strategy intending to prevent NCDs that are currently suspected to be involved in oral diseases.

There is only single study based on the database for the National Health Insurance Service-National Sample Cohort (NHIS–NSC) in South Korea that has identified associations between PD and lifestyle-related comorbidities including cardiovascular disease, hypertension, diabetes mellitus, rheumatoid arthritis, erectile dysfunction, osteoporosis, and obesity; no previous research has used the database for the National Health Insurance Service–Health Examinee Cohort (NHIS–HEC) study released in 2016.^[12,13]^ The purpose of the present study was, therefore, to determine the incidence of PD and its associations with major NCDs including hypertension, diabetes mellitus, osteoporosis, cerebral infarction, angina pectoris, myocardial infarction, and obesity using the database for the nationwide population-based NHIS–HEC.

## 2 Methods

### 2.1 Study design and data collection

More than 97% of the Korean population was covered by National Health Insurance (NHI) in 2015. Those eligible for NHI are directed by the NHIS to undergo regular health checkup examinations in order to maintain and promote their health and thereby reduce their respective long-term insurance payouts. These regular health checkup examinations are classified into general health examinations (primary and secondary), health examinations of the life-span transition period (primary and secondary, for people aged 40 years and 66 years), cancer examinations (gastric, breast, colorectal, cervical, and liver cancer), and infant/child health examinations. Individuals who qualify as being insured for health examinations may undergo regular health checkups in medical institutions appointed by the NHIS.

This study selected the 51.5 million examinees (aged 40–79 years at the end of December 2002) who underwent regular health checkup examinations during 2002 and 2003 as the entire population, from which 10% (n = 514,866) were sampled by simple stratification. Since the beneficiaries of the medical aid program (MAP) in 2002 were not eligible to undergo a general health examination, they were excluded from the population selected to sample the subjects for 2002. The database included the following parameters: qualification parameters for checkups (sex, age, insurance status, residence area, and health status), household income, history of attending medical institutions, and records of health examinations including body mass index (BMI), systolic blood pressure (SBP), diastolic blood pressure (DBP), fasting blood glucose (FBG), total cholesterol (TC), hemoglobin, aspartate aminotransferase (AST), alanine aminotransaminase (ALT), and r-glutamyltranspeptidase (r-GTP). These parameters were collected for 12 years (2002–2013) and rearranged to prevent the identification of individuals for the cohort study.

This study conformed to the STROBE guidelines for reporting observational studies (www.strobe-statement.org) and was approved by the Institutional Review Board, Daejeon Dental Hospital, Wonkwang University (approval no. W1611/001–001).

### 2.2 Identification of non-communicable diseases

Principal and subprincipal diagnostic and prescription codes were collected for each patient based on the databases of the NHIS and HIRA. The following NCDs were diagnosed by physicians or other medical professionals during visits to out- or in-patient clinics from 2002 to 2013: hypertension (Korean Classification of Diseases, 6th revision [KCD-6], codes I10 and I15; corresponding to the International Classification of Disease, 10th revision [ICD-10], codes I10 and I15), diabetes mellitus (KCD-6 codes E10–E14, corresponding to ICD-10 codes E10–E14), osteoporosis (KCD-6 codes M08–M82, corresponding to ICD-10 codes M08–M82), cerebral infarction (KCD-6 codes I63–I66, corresponding to ICD-10 codes I63–I66), angina pectoris (KCD-6 code I20, corresponding to ICD-10 code I20), myocardial infarction (KCD-6 code I21–I22, corresponding to ICD-10 code I21–I22), and obesity (KCD-6 code E66, corresponding to ICD-10 code E66). The date of the initial NHIS and HIRA claims related to NCDs was assigned as the index date in the analysis, and we excluded patients who had already experienced NCDs based on their responses to a self-reported questionnaire in the health checkup examination. To increase the validity of the assessment of NCD diagnoses, we only included patients who had been diagnosed at least twice with a specific NCD-related disorder from 2002 through 2013.

### 2.3 Identification of patients with periodontal disease

In order to improve diagnostic accuracy of PD in patients, oral examination data has been used besides diagnostic code (KCD-6 codes K05.2–K05.6, corresponding to ICD-10 codes K05.2–K05.6) which has been confirmed before index date. PD was diagnosed clinically in oral checkup examinations based on assessments of missing teeth, gingival/periodontal inflammation (swelling and redness), and the deposition of calculus in accordance with criteria of the Centers for Disease Control and Prevention/American Academy of Periodontology by a general dentist or a periodontitis in the biannual NHIS programme.^[14]^

### 2.4 Parameters in the health checkup examinations

The health checkup examinations in the NHIS–HEC included a self-reported questionnaire, anthropometric measurements, and blood laboratory measurements. Subjects who responded in self-reported questionnaires that they were currently not smoking, or had smoked fewer than 100 cigarettes were classified as nonsmokers, while the other subjects were classified as smokers. Weight and height were measured without shoes. BMI was calculated by dividing the weight by height squared. SBP and DBP were measured by medical personnel or with an automatic oscillometry device. FBG, TC, hemoglobin, AST, ALT, and r-GTP levels were assessed in blood samples drawn from the subjects. Subjects with SBP < 60 mmHg and >400 mmHg, DBP <30 mmHg and >250 mmHg, FBG <25 mg/dL and >999 mg/dL, TC <40 mg/dL and >999 mg/dL, hemoglobin >25.0 g/dL, and AST, ALT, and r-GTP levels >999 IU/L were assumed to be either unknown or missing values, and, therefore, were excluded from the study sample.

### 2.5 Definition of sociodemographic factors

The sociodemographic factors were collected from the NHIS–HEC and categorized as follows using random stratified analysis by the NHIS Big Data Steering Department: sex (2 groups), age (8 groups classified into those aged 40–69 years in 10-year intervals, and those >70 years old), monthly household income (41 groups classified into quintiles: MAP beneficiaries were in the first-quintile group), insurance status (2 groups: self-employed and employees), residence area (2 groups based on metropolitan [≥1,000,000 residents] or not metropolitan [<1,000,000 residents] living), health status (3 groups classified into healthy, minor disability, and major disability based on the Handicapped Welfare Law), and smoking status.

### 2.6 Statistical analysis

We used chi-squared, univariate, and multivariate logistic regression analyses to determine the incidence of PD and its associations with major NCDs for the entire enrolled population. Odds ratios (ORs) and 95% confidence intervals (CIs) were calculated, and a *P* value of less than .05 was considered to be statistically significant. All statistical analyses were performed by the Department of Health Insurance Research, Ilsan Hospital, NHIS using the Statistical Analysis System (version 9.2, SAS Institute, Cary, NC).

## 3 Results

### 3.1 Baseline characteristics and incidence of periodontal disease

**Figure 1:**
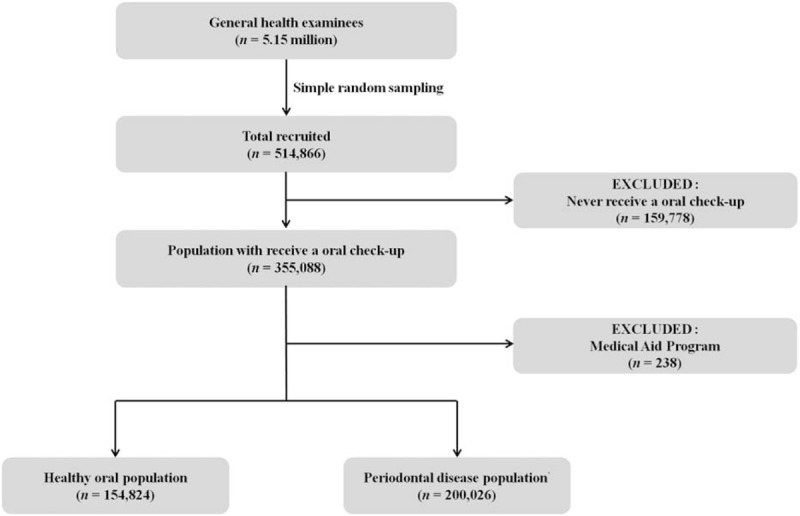
Flow chart of the inclusion and exclusion of participants in the National Health Insurance Service–Health Examinee Cohort during 2002 to 2013.

Figure 1 shows a flow chart of the inclusion and exclusion process for participants in this study. Among the 514,866 South Korean subjects originally included, 354,850 (68.9%) were recruited: 199,886 men (56.3%) and 154,964 women (43.7%). Those aged 40 to 59 years (n = 178,670) accounted for 50.4% of the surveyed subjects, while 127,465 (35.9%) were in the fifth quintile of household income, 233,385 (65.8%) were in the NHIS (employees), 218,276 (61.5%) did not live in metropolitan areas, and 353,251 (99.5%) had a healthy status. Among the 354,850 subjects, 200,026 (56.4%) patients with PD were recruited, consisting of 121,236 men (60.6%) and 78,790 women (39.4%). Those aged 40 to 59 years (n = 106,148) accounted for 53.1% of the surveyed subjects, while 75,232 (37.6%) were in the fifth quintile of household income, 138,273 (69.1%) were in the NHIS (employees), 120,092 (60.0%) did not live in metropolitan areas, and 199,292 (99.6%) had a healthy status (Table 1).

**Table 1:**
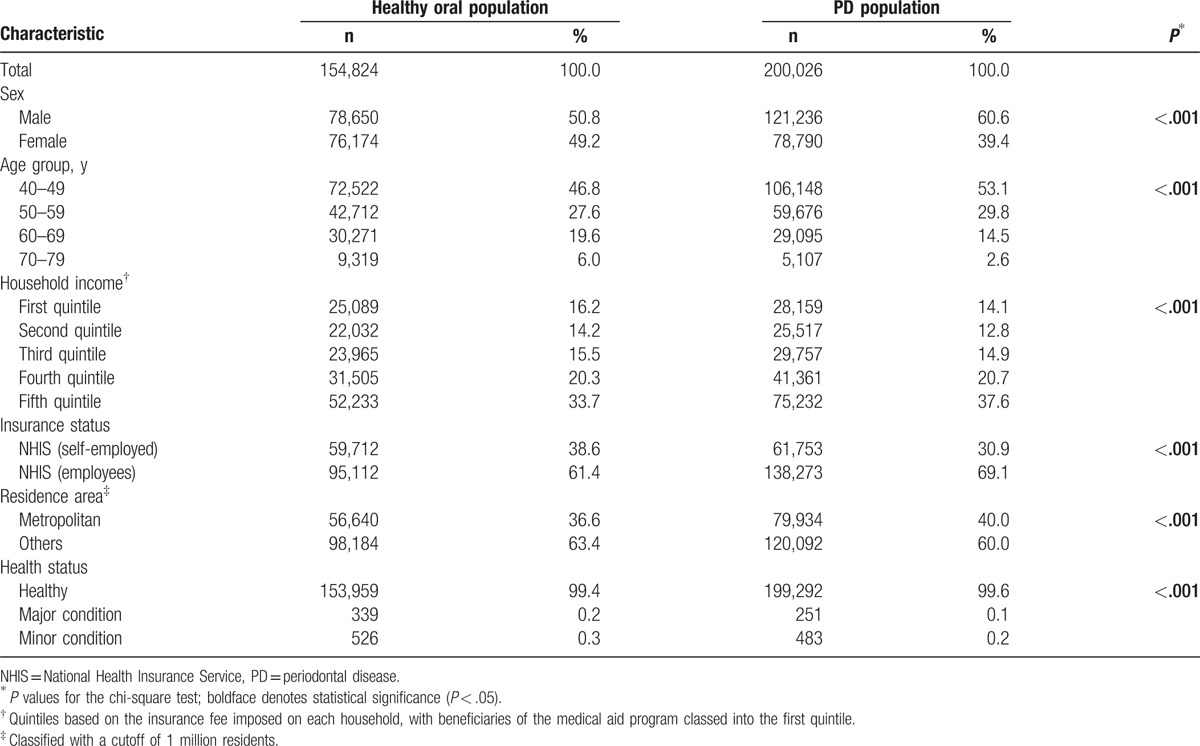
Baseline characteristics of participants in the National Health Insurance Service–Health Examinee Cohort Study (NHIS–HEC).

### 3.2 Results of the health checkup examinations

The numbers of the subjects identified as smokers, nonsmokers, and not providing responses in health checkup examinations were 81,454 (23.1%), 256,105 (72.2%), and 16,900 (4.7%), respectively. The mean levels of BMI, DBP, TC, hemoglobin, AST, ALT, and r-GTP were higher in the PD population than in those with a healthy oral status, while SBP and FBG were lower in the PD population. Smoking status, BMI, SBP, FBG, TC, hemoglobin, ALT, and r-GTP differed significantly between the healthy oral and PD populations (Table 2).

**Table 2:**
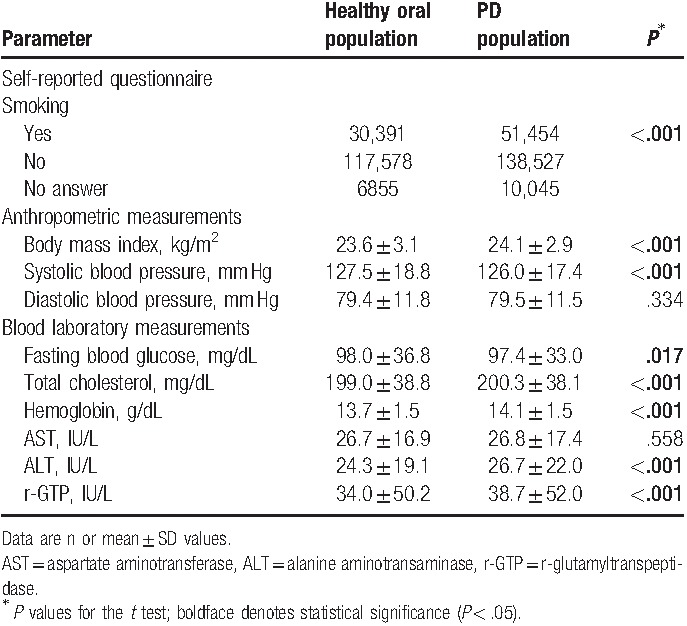
Results of health checkup examinations.

### 3.3 Associations of non-communicable diseases with periodontal disease

As indicated in Table 3, the univariate logistic regression analysis showed that PD was significantly positively related to diabetes mellitus (OR = 1.02, 95% CI = 1.01–1.04, *P* < .001), and significantly negatively related to hypertension (OR = 0.95, 95% CI = 0.93–0.96, *P* < .001), osteoporosis (OR = 0.77, 95% CI = 0.76–0.78, *P* < .001), and cerebral infraction (OR = 0.84, 95% CI = 0.83–0.86, *P* < .001).

**Table 3:**
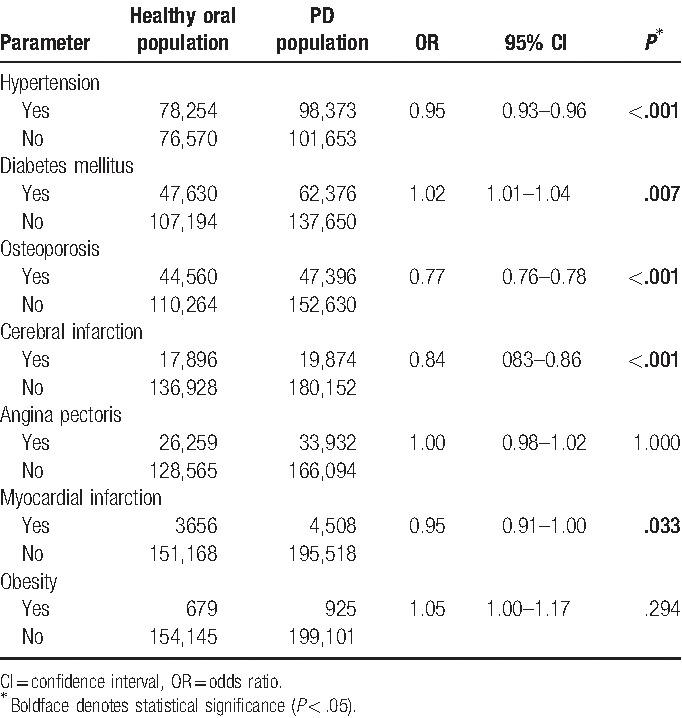
Association of non-communicable diseases with periodontal disease in univariate analyses.

In the multivariate logistic regression analysis with adjustment for sex, age, household income, insurance status, residence area, health status, and smoking status, PD was significantly positively related to hypertension (OR = 1.04, 95% CI = 1.01–1.07, *P* < .014), diabetes mellitus (OR = 1.16, 95% CI = 1.12–1.20, *P* < .001), osteoporosis (OR = 1.22, 95% CI = 1.18–1.27, *P* < .001), angina pectoris (OR = 1.22, 95% CI = 1.17–1.27, *P* < .001), and obesity (OR = 1.30, 95% CI = 1.04–1.63, *P* = .022). PD was significantly negatively related to myocardial infarction (OR = 0.88, 95% CI = 0.81–0.97, *P* = .007) and was not significantly related to cerebral infraction (OR = 0.98, 95% CI = 0.94–1.03, *P* = .418; Table 4).

**Table 4:**
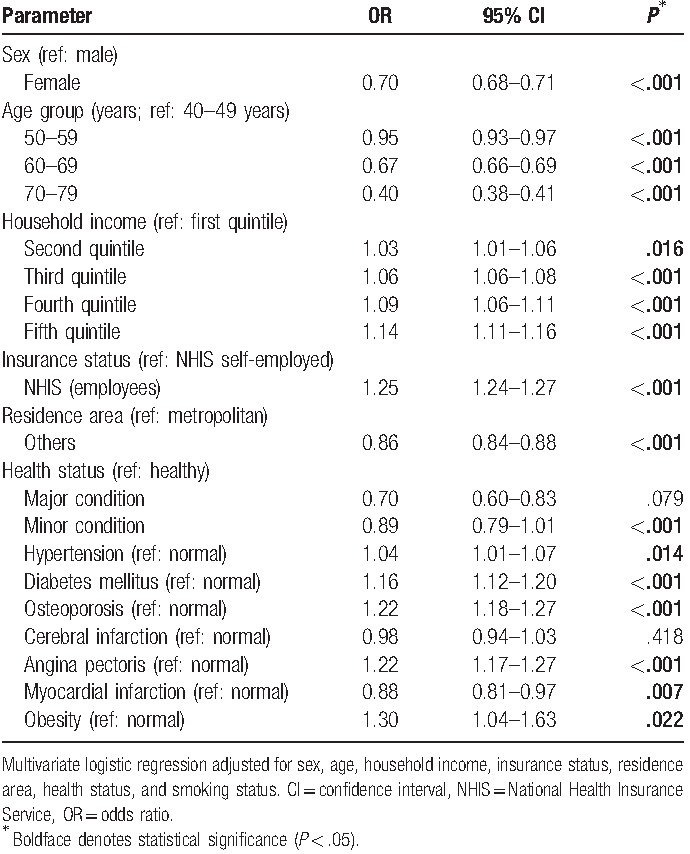
Association of non-communicable diseases with periodontal disease in multivariate analyses.

## 4 Discussion

To our knowledge, this study is the first to provide evidence for the links between PD and major NCDs using the nationwide database of the population-based NHIS–HEC. The results revealed that PD was diagnosed in 200,026 (56.3%) of 355,088 subjects who underwent health checkup examinations. Multivariate logistic regression analyses with adjustment for sociodemographic factors and smoking status revealed that hypertension, diabetes mellitus, osteoporosis, angina pectoris, and obesity were significantly positively associated with PD, whereas PD was not significantly associated with cerebral or myocardial infarction. These findings are consistent with the results obtained in a previous study that explored the correlations between PD and lifestyle-related comorbidities in the Korean population using the NHIS–NSC.^[12]^

The health checkup examination included basic blood tests that revealed significant differences between the healthy oral and PD populations in BMI (*P* < .001), SBP (*P* < .001), FBG (*P* = .017), TC (*P* < .001), hemoglobin (*P* < .001), ALT (*P* < .001), and r-GTP (*P* < .001) levels. Although the mean values of BMI, DBP, TC, hemoglobin, AST, ALT, and r-GTP were higher in patients with PD than in those with a healthy oral status, the differences in these levels between the 2 groups were within the normal ranges and medically insignificant.^[15]^ Changes in dietary habits, decreased physical activity, and increased stress are related to metabolic disorders such as severe obesity, hypertension, diabetes mellitus, and hyperlipidemia, and have been reported to be relevant to the increasing prevalence of PD.^[16]^ The common biological mechanisms underlying both PD and metabolic disorders have yet to be clarified, but the involvement of PD (a chronic inflammatory disease) in various immune reactions of the host and the resulting effects on glucose and lipid metabolism have been established in several studies.^[17,18]^

The present multivariate logistic regression analyses only showed a significant positive association of PD with angina pectoris (OR = 1.22, 95% CI = 1.17–1.27, *P* < .001) in major cardiovascular disease; the negative association with both cerebral infarction (OR = 0.98, 95% CI = 0.94–1.03, *P* = .418) and myocardial infarction (OR = 0.88, 95% CI = 0.81–0.97, *P* = .007) could be attributed to the use of the NHIS–HEC database, wherein voluntary health examinees were included by simple sampling. Since cerebral and myocardial infarctions are associated with high mortality rates and serious sequelae in the middle-age and elderly population, patients suffering these diseases might have been excluded from the NHIS–HEC database. It was suspected that the patients with PD with these 2 diseases were unlikely to visit dental clinics to be diagnosed with PD, resulting in the lower ORs in this study.^[19]^ Several research studies have suggested a connection between cardiovascular disease and PD, they share certain physiological and sociodemographic risk factors, such as sex, smoking, obesity, stress, lower income, and senility.^[20,21]^ The presence of PD may lead to transient or intermittent cardiac bacteremia by periodontal pathogens such as *Porphyromonas gingivalis* (*P. gingivalis*), *Aggregatibacter actinomycetemcomitans*, and *Fusobacterium nucleatum*. In particular, the introduction of *P. gingivalis* into the bloodstream can represent a source of infection and it underlies systemic inflammation, endothelial dysfunction, and isoproterenol-induced cardiac hypertrophy.^[22,23]^

Diabetes mellitus and hypertension are reportedly important risk factors for PD; therefore, controlling these 2 factors is likely to be critical in the prevention and treatment of adult PD.^[24,25]^ In this study, the OR for the association of PD with diabetes mellitus in the multivariate analysis was 1.16 (CI = 1.12–1.20, *P* < .001), while that for hypertension was 1.04 (CI = 1.01–1.07, *P* = .014). The association between diabetes mellitus and PD can be explained as a manifestation of systemic inflammation and the corresponding mechanisms of insulin sensitivity and glucose dynamics.^[26]^ The increased severity or chronicity of PD increases the insulin resistance and aggravates glycemic control.^[27]^ Many studies have yielded evidence for a link between diabetes mellitus and essential hypertension via hyperinsulinemia.^[28,29]^ Therefore, the interrelatedness of diabetes mellitus, hypertension, and PD may affect the manifestation of the disease.

The association of PD with obesity exhibited the highest OR (1.30, CI = 1.04–1.63, *P* = .022) in the multivariate analysis. Obesity is associated with a higher fat level, and it has recently been recognized as a both major risk factor for NCDs and a pathological disease that affects individuals irrespective of age.^[29]^ Obesity causes increased oxidative stress which leads not only to local and systemic endothelial dysfunction, but also to an early connection between PD and obesity.^[30]^ A recent systematic review found a fixed-effects summary OR of 1.35 (CI = 1.23–1.47, *P* < .005), which is similar to that obtained in the present study. In particular, a significantly higher association among younger adults, women, and nonsmokers was found.^[31]^ Conversely, another 5-year cohort study examining the association between PD and obesity found a higher prevalence in men.^[32]^ Moreover, another study found greater periodontal destruction including clinical attachment loss in patients with a higher BMI, while yet another found no significant association between PD and a BMI of >30 kg/m^2^.^[33,34]^ While any definitive relationships between the clinical periodontal parameters associated with PD and obesity remain to be clarified, several reports on the potential effects of increased leptin and interleukin (IL)-6 levels on PD and obesity must be taken into consideration.^[35]^

Many previous studies have found no association between PD and osteoporosis due to either an inadequate number of subjects or the included subjects being limited by old age or being postmenopausal women.^[36,37]^ A recent nationwide population cohort study that controlled for sociodemographic and economic factors found a significant association between PD and osteoporosis (OR = 1.96, 95% CI = 1.17–3.26), and the present study conducted with adult man and woman subjects older than 40 years also found a significant association in the multivariate analysis (OR = 1.22, 95% CI = 1.18–1.27, *P* < .001).^[38,39]^ Osteoporosis is a disease with shared risk factors such as age, smoking, alcohol consumption, diabetes mellitus, obesity, and common clinical features of bone loss and destruction.^[40]^ Patients with osteoporosis show increased activity of inflammatory cytokines such as IL-1 and IL-6, tumor necrosis factor-α, and increased activity of osteoclasts in alveolar bone.^[41]^ Most longitudinal and cross-sectional studies demonstrated the relationship between PD and osteoporosis based on radiographic measurements and clinical parameters.^[42]^ The underlying mechanisms suspected include the disruption of both homeostatic bone metabolism and inflammation resolution.^[42]^ Osteoporosis, therefore, needs to be considered a possible risk factor for the progression and aggravation of PD.^[43,44]^

This study was subject to some important limitations. First, as mentioned above, since only the diagnostic and corresponding prescription codes that are registered in the NHIS were used in the present analyses, databases of voluntary non-reimbursable treatments or of the MAP that are not registered in the NHIS were neglected. Moreover, the personal health checkup examination database was also excluded. The MAP is planned to be included in the NHIS–HEC in the future, which is expected to decrease selection bias. Second, since the NHIS–HEC was prepared by diverse medical and dental specialties under NHIS supervision, there is a possibility of inconsistency between cases with identical diagnoses. In particular, since PD can be diagnosed either with or without panoramic/periapical radiographic images, this could increase the likelihood of inconsistency in such diagnoses. Nevertheless, difficulties due to such problems were avoided in the present study by employing the NHIS–HEC data on national oral checkup examinations, which allowed patients with PD to be accurately distinguished.

This study has demonstrated that the presence of PD is associated with a significantly elevated risk of NCDs in the Korean adult population, especially obesity, osteoporosis, and angina pectoris. Additional studies are required to confirm this association and to establish in detail the role of the inflammatory pathway in the pathogenesis of periodontitis as a triggering and mediating mechanism.

## Acknowledgments

The used NHIS–HEC data (NHIS-2017–2–309) were supplied by the NHIS.
